# Conditioned media from human macrophages of M1 phenotype attenuate the cytotoxic effect of 5-fluorouracil on the HT-29 colon cancer cell line

**DOI:** 10.3892/ijo.2014.2696

**Published:** 2014-10-07

**Authors:** ALEXANDER HEDBRANT, ANN ERLANDSSON, DICK DELBRO, JONNY WIJKANDER

**Affiliations:** 1 Department of Health Sciences, Karlstad University, Karlstad, Sweden; 2 School of Health and Medical Sciences, Örebro University, Örebro, Sweden

**Keywords:** chemotherapy, 5-fluorouracil, colon cancer cell line, HT-29, CACO-2, M1 macrophages, M2 macrophages, cell cycle

## Abstract

Resistance of tumor cells to chemotherapy, such as 5-fluorouracil (5-FU), is an obstacle for successful treatment of cancer. As a follow-up of a previous study we have investigated the effect of conditioned media (CM) from macrophages of M1 or M2 phenotypes on 5-FU cytotoxicity on the colon cancer cell lines HT-29 and CACO-2. HT-29 cells, but not CACO-2 cells, having been treated with a combination of M1 CM and 5-FU recovered their cell growth to a much larger extent compared to cells having been treated with 5-FU alone when further cultured for 7 days in fresh media. M1 CM treatment of HT-29, but not CACO-2 cells, induced cell cycle arrest in the G_0_/G_1_ and G_2_/M phases. 5-FU treatment induced accumulation of cells in S-phase in both HT-29 and CACO-2 cells. This accumulation of cells in S-phase was attenuated by combined M1 CM and 5-FU treatment in HT-29 cells, but not in CACO-2 cells. The mRNA expression of cell cycle regulatory proteins and 5-FU metabolic enzymes were analyzed in an attempt to find possible mechanisms for the M1 CM induced attenuation of 5-FU cytotoxicity in HT-29. Thymidylate synthetase (TS) and thymidine phosphorylase (TP) were found to be substantially downregulated and upregulated, respectively, in HT-29 cells treated with M1 CM, making them unlikely as mediators of reduced 5-FU cytotoxicity. Among cell cycle regulating proteins, p21 was induced in HT-29 cells, but not in CACO-2 cells, in response to M1 CM treatment. However, small interfering RNA (siRNA) knockdown of p21 had no effect on the M1 CM induced cell cycle arrest seen in HT-29 and neither did it change the growth recovery after combined treatment of HT-29 cells with M1 CM and 5-FU. In conclusion, treatment of HT-29 cells with M1 CM reduces the cytotoxic effect of 5-FU and this is mediated by a M1 CM induced cell cycle arrest in the G_0_/G_1_ and G_2_/M phases. So far, we lack an explanation why this action is absent in the CACO-2 cells. The current findings may be important for optimization of chemotherapy in colon cancer.

## Introduction

Colorectal cancer (CRC) is one of the most prevalent cancers in the Western world and it is the second cause of cancer deaths in Europe (1). In solid cancers including CRC, immune cells in the tumor stroma play an important role in cancer progression, participating in the regulation of processes such as cancer cell proliferation, immune suppression and invasion/metastasis (2–4). In the stroma of CRC, the tumor-associated macrophages (TAMs) are an important cell type suggested to modulate cancer progression (5–7).

Macrophages can display different phenotypes depending on their microenvironment and are classified as classically activated pro-inflammatory M1 macrophages, or alternatively activated anti-inflammatory M2 macrophages (8–10). The M1 phenotype results from the activation by lipopolysaccharide (LPS) and/or interferon-γ (IFN-γ) and release pro-inflammatory factors such as tumor necrosis factor-α, interleukin (IL)-6, and IL-12, and cytotoxic substances such as reactive oxygen and nitrogen species (11). The M2 phenotype, on the other hand results from a polarization by cytokines such as IL-4 or IL-13, and release anti-inflammatory cytokines, e.g., IL-10 and transforming growth factor-β, and angiogenic factors such as vascular endothelial growth factor (12–14). Due to the notion that the macrophages display a broad functional spectrum as well as an ability to change function depending on the microenvironment the effect of TAMs on cancer progression is difficult to predict, and results regarding a relationship between TAM levels and prognosis in CRC are contradictory (6,7,15–18). Such conflicts could, at least in part, be explained by how these macrophages have been polarized by the tumor microenvironment (19). Moreover, presence of TAMs in the tumor microenvironment might not only affect tumor progression, but also the response to treatments like e.g., chemotherapy or radiation therapy (10).

Colon cancer is always treated by surgery and, depending on tumor stage, is often supplemented with adjuvant 5-fluorouracil (5-FU) based chemotherapy. Since 5-FU has limited antitumor activity on its own, combinational therapy, such as the FOLFOX treatment is often used, which also includes oxaliplatin and leucovorin, to enhance the antitumor activity of the treatment (20,21).

5-FU is a chemotherapeutic agent classified as an anti-metabolite that primarily acts by the irreversible inhibition of thymidylate synthetase (TS) leading to deoxythymidine monophosphate (dTMP) shortage and non-functional DNA synthesis. Thereby, 5-FU is primarily an S-phase specific drug that acts on actively proliferating cells. Therefore, factors regulating cell proliferation or the cell cycle can modulate 5-FU cytotoxicity (22). Another factor that can affect 5-FU cytotoxicity is the expression levels of various enzymes responsible for intracellular metabolism of 5-FU, which can either result in the primary active substance, fluorodeoxyuridine monophosphate (FdUMP) or the non-active substance, dihydrofluorouracil (23,24). We recently reported that M1 macrophages can reduce proliferation and induce cell cycle arrest of colon cancer cells, as investigated with the HT-29 cell line, *in vitro* (25). As a follow-up, the aim of the current study was to examine whether conditioned media (CM) from human M1 or M2 macrophages could affect the efficacy of 5-FU treatment of colon cancer cells. Specifically, we investigated effects on proliferation, cell cycle distribution and expression of cell cycle regulating genes and 5-FU metabolic genes in the colon cancer cell lines HT-29 and CACO-2.

## Materials and methods

### Cell culture

The colon cancer cell lines, HT-29 and CACO-2, were purchased from DSMZ (Braunschweig, Germany). Each cell line was cultured in RPMI-1640 (RPMI; Life Technologies, Carlsbad, CA, USA) supplemented with 2 mM L-glutamine, 100 U/ml penicillin and 100 μg/ml streptomycin (Life Technologies) with 10% heat-inactivated fetal calf serum (FCS) (Thermo Fisher Scientific, Inc., Waltham, MA, USA) and 10 mM HEPES. Both cell lines were grown at 37°C in a humidified atmosphere and 5% CO_2_.

**Figure 1:**
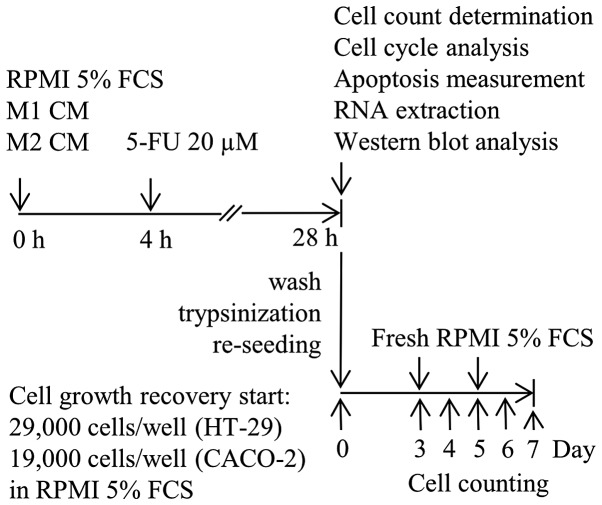
Treatment schedule for experiments performed with HT-29 or CACO-2 cells in the present investigation. Cells were treated as indicated, and in case of combined treatment, 5-fluorouracil (5-FU) (20 μM) was added after 4 h. All experiments were terminated after a total time of 28 h. For cell growth recovery experiments, instead of termination, the cells were washed with RPMI, detached by trypsinization, counted, and re-seeded in RPMI 5% fetal calf serum (FCS), indicated as day 0. Each day between day 3 and 7, duplicate wells of each treatment were terminated for assessment of cell growth recovery.

For all experiments, 29,000 HT-29 cells/well or 19,000 CACO-2 cells/well were seeded onto 24-well plates (Greiner Bio-One GmbH, Frickenhausen, Germany) in 0.5 ml RPMI 10% FCS plus 10 mM HEPES and cultured for 3 days. Thereafter, cells were treated with M1 or M2 macrophage CM (for preparation see below) or 5-FU, alone or in combination, according to the schedule shown in Fig. 1.

### Isolation of human monocytes and differentiation to macrophages

Buffy coats from healthy blood donors were obtained from the division of Clinical Immunology and Transfusion Medicine, Uppsala University Hospital (Uppsala, Sweden), and monocytes were isolated by gradient centrifugation and allowed to differentiate into macrophages with macrophage colony-stimulating factor (M-CSF) treatment for 6 days, as described previously (25). After macrophage differentiation, the macrophages were further differentiated into M1 macrophages through the addition of 100 ng/ml LPS (Sigma-Aldrich, St. Louis, MO, USA) plus 20 ng/ml IFN-γ for 48 h or M2 macrophages through the addition of 20 ng/ml IL-4 plus 20 ng/ml IL-13 (all from R&D Systems, Minneapolis, MN, USA) for 48 h. The differentiated M1 and M2 macrophages [the phenotypes were characterized as described previously (25)] were washed twice with PBS and were cultured for another 48 h in RPMI 5% FCS (without either IFN-γ/LPS or IL-4/IL-13) to generate M1 and M2 CM. The collected CM was centrifuged to remove cell debris and stored in aliquots at −20°C.

### Proliferation studies and cell growth recovery assessment

HT-29 or CACO-2 cells were cultured as described above in the cell culture section and treated as described in Fig. 1 and counted in a hemocytometer. For growth recovery assessment, treated cells were washed, detached by trypsinization, counted in a hemocytometer and subsequently re-seeded at 29,000 HT-29 cells/well or 19,000 CACO-2 cells/well for each treatment onto 24-well cell culture plates (Greiner Bio-One GmbH) in 0.5 ml RPMI 5% FCS. Cells were thereafter counted in a hemocytometer at day 3–7 after treatment. Media renewal was done at day 3 and 5.

### Cell cycle analysis

HT-29 or CACO-2 cells were cultured as described above and were treated as described in Fig. 1. Subsequently, the cells were detached by trypsinization and were pooled with their corresponding culture media possibly containing loose cells. The cells were washed with PBS containing 1% bovine serum albumin (PBS/BSA) and were resuspended in 450 μl ice-cold PBS/BSA prior to fixation in 5 ml ice-cold 70% ethanol. Samples were stored at −20°C until analysis.

Before analysis, Triton X-100 was added to a final concentration of 0.1% (v/v) and the cells were incubated for 10 min at 6°C. Next, the cells were washed twice with PBS/BSA and were resuspended in 1 ml PBS/BSA containing 0.1% Triton X-100, 50 μg/ml propidium iodide and 200 μg/ml RNase A (both from Sigma-Aldrich). Samples were incubated at room temperature for 45 min in the dark prior to analysis on a FACSCalibur (BD Biosciences, San Jose, CA, USA) flow cytometer. Cell cycle distributions were calculated using the ModFit LT software v.3.1 (Verity Software House, Inc., Topsham, ME, USA).

### RNA extraction and cDNA synthesis

HT-29 and CACO-2 cells were cultured as described above and were treated as described in Fig. 1. Cells were detached by trypsinization and total RNA extracted using the RNeasy Mini kit (Qiagen, Hilden, Germany) according to manufacturer’s instructions. RNA quantity and purity, respectively, was determined by measuring the absorbance at 260 nm and the 260/280 nm ratio, respectively, in a nanoquant plate analyzed with the infinite M200 Pro plate reader (Tecan, Männedorf, Switzerland). cDNA was transcribed from 1 μg total RNA using the High-Capacity cDNA Reverse Transcription kit (Applied Biosystems, Foster City, CA, USA) according to manufacturer’s instructions.

### Quantitative PCR (qPCR)

qPCR was run on a StepOnePlus Real-Time PCR using Power SYBR-Green Master Mix (both from Applied Biosystems) with a reaction volume of 25 μl including 1 μl cDNA and 200 nM of each primer (Table I for sequences). All reactions were run in triplicates. Fold change of treated sample vs. untreated control was calculated with REST2009 software (Qiagen) (26) using both GAPDH and POLR2F as housekeeping genes for HT-29 samples and GAPDH, POLR2F, RPL37A and β-actin for CACO-2 samples. The efficiency of primers was calculated using LinRegPCR software (27). The size of amplified qPCR products was validated by agarose gel-electrophoresis for all primer pairs.

### Apoptosis measurement

HT-29 and CACO-2 cells were cultured as described above and were treated as described in Fig. 1. Cells were detached by trypsinization and pooled with their corresponding cell culture media possibly also containing floating cells, were centrifuged at 300 × g for 5 min and then resuspended in 1% paraformaldehyde in PBS. Cell suspensions were incubated on ice for 45 min. Next, cells were washed twice with 5 ml PBS and were resuspended in 450 μl ice-cold PBS prior to cell fixation in 5 ml ice-cold 70% ethanol. Fixed cells were stored at −20°C until apoptosis measurements were done using a terminal deoxynucleotidyl transferase dUTP nick end labeling (TUNEL) kit (Phoenix Flow Systems, San Diego, CA, USA) according to manufacturer’s instructions. Cell apoptosis was analyzed on a FACSCalibur flow cytometer and acquired data were analyzed with Cell Quest v.3.3 (both from BD Biosciences).

### p21 siRNA gene knockdown

HT-29 cells were cultured as described above. After 2 days of culture, the culture medium was changed to RPMI 5% FCS and small interfering RNA (siRNA) against p21 (Silencer Select ID s415) (30 nM) or negative scramble sequence (Silencer Select Negative Control no. 1) (30 nM) were added together with the lipofectamine RNAiMax Transfection Reagent dissolved in Opti-MEM medium according to the manufacturer’s instructions (all from Life Technologies). Following a 24 h transfection, the transfection media were removed and cells were treated as described in Fig. 1 and effects of p21 siRNA knockdown were analysed with respect to cell count, cell growth recovery, cell cycle distribution, and p21 expression.

### Immunoblotting

For whole cell lysates, HT-29 and CACO-2 cells were cultured as described above. Samples from siRNA experiments were treated as described in the p21 siRNA knockdown section. For non-siRNA experiments, HT-29 or CACO-2 cells were treated for 28 h with RPMI 5% FCS or M1 CM. After treatment, the cells were detached by trypsinization, counted, pelleted, and were resuspended in 1 μl/10,000 cells of Laemmli sample buffer with protease inhibitors [20 μg/ml aprotinin, 10 μg/ml leupeptin, 5 mM phenylmethylsulfonyl fluoride (all from Sigma-Aldrich)]. Lysed samples were homogenized through a 21 gauge syringe needle, boiled for 5 min and were stored in aliquots at −20°C until analysis.

Samples (20 μl) were subjected to SDS-PAGE (12% acrylamide gel) and proteins were transferred onto a PVDF membrane (Bio-Rad, Hercules, CA, USA). Blocking was performed for 1 h at room temperature with 5% BSA in TBS 0.1% Tween-20 (TBS-T). Membranes were incubated overnight at 4°C with p21 antibodies (12D1 rabbit monoclonal antibody; Cell Signaling Technology, Inc, Danvers, MA, USA) diluted 1:1,000 in blocking solution. The secondary antibody (goat anti-rabbit IgG-HRP, Sc-2004; Santa Cruz Biotechnology, Inc., Dallas, TX, USA) was diluted 1:15,000 in TBS-T and was applied for 1 h at room temperature. Signals were developed using SuperSignal West Pico Chemiluminescent Substrate (Thermo Fisher Scientific, Inc.) and light intensities were detected by exposure to Amersham Hyperfilm ECL (GE Healthcare, Little Chalfont, UK). After detection of p21, membranes were stripped using Restore Western Blot Stripping Buffer (Thermo Fisher Scientific, Inc.) and were incubated with β-actin antibody as loading control (N-21 rabbit polyclonal antibody; Santa Cruz Biotechnology, Inc.) diluted 1:500 in blocking solution.

### Statistics

All the results are presented as mean value ± standard deviation (SD). Two-sided Student’s t-test was used for the statistical analyses. All experiments with macrophage CM were performed with at least three different macrophage batches generated from different donors.

## Results

### CM from macrophages of M1 phenotype attenuates 5-FU mediated growth inhibition of HT-29 cells, but not of CACO-2 cells

**Figure 2:**
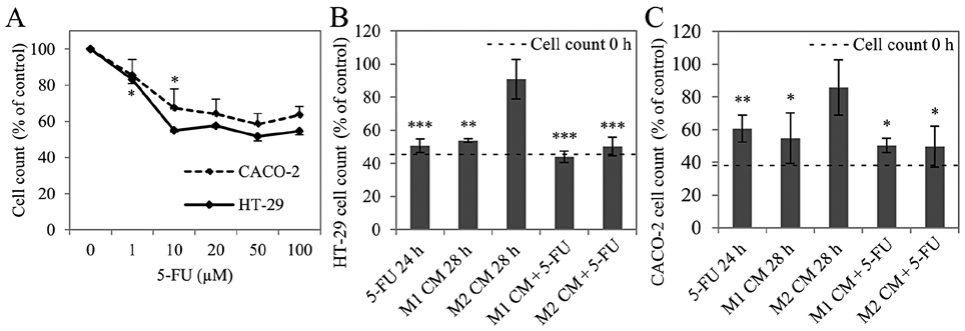

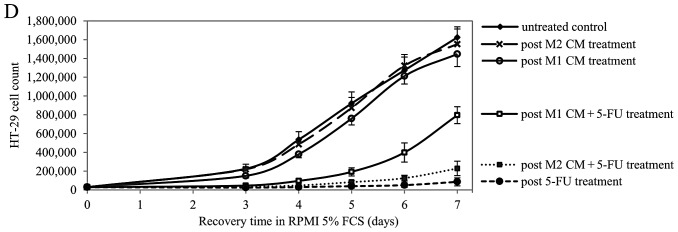

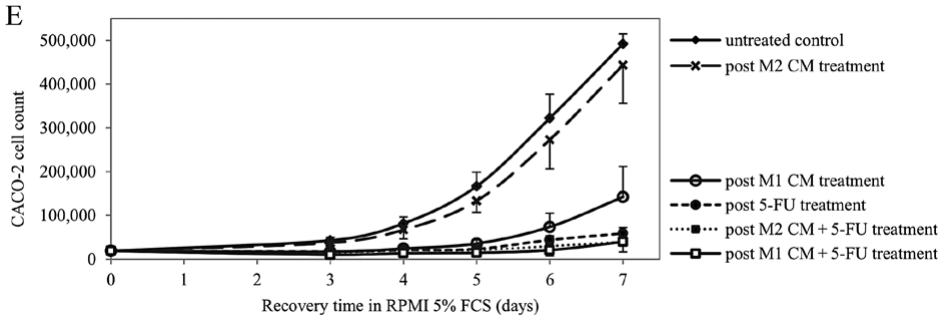
Effect of 5-fluorouracil (5-FU) and conditioned media (CM) from macrophages of M1 or M2 phenotype on the proliferation of (A and B) HT-29 cells and (A and C) CACO-2 cells. Cell growth recovery after indicated treatments of (D) HT-29 cells and (E) CACO-2 cells. Cells were treated as described in Fig. 1 (Materials and methods section). The growth recovery experiments were conducted on treated cells detached by trypsinization and were re-seeded (HT-29: 29,000 cells/well, CACO-2: 19,000 cells/well) in RPMI 5% fetal calf serum (FCS) and counted day 3–7 after re-seeding. Results are shown as mean value ± standard deviation (SD). (A) n ≥ 3 independent experiments. (B and D) At least four independent experiments with four different macrophage batches from different donors were used. (C and E) At least three independent experiments with three different macrophage batches from different donors were used. The dotted line in (B and C) indicate cell number at the start of treatment. ^*^P<0.05, ^**^p<0.01, ^***^p<0.001.

Dose-response analysis of a 24 h 5-FU treatment of HT-29 or CACO-2 cells revealed similar inhibition of growth for either cell line with a partial inhibition at 1 μM 5-FU (cell count reduced to about 80% of control) and reaching a plateau at about 10 μM 5-FU (cell count reduced to about 60% of control) (Fig. 2A). For all further experiments 20 μM 5-FU was chosen for treatment of either cell line.

In accordance with previously published results (25), M1 CM induced a strong reduction in growth of both HT-29 and CACO-2 cells when treated for 28 h whereas treatment with M2 CM had no significant effect (Fig. 2B and C). No apparent additive effect in reduction of cell growth could be seen when cells were treated with both 5-FU and M1 CM (4 h with M1 CM + 24 h in combination with 5-FU). To further evaluate the effect of these treatments on cell growth, were-seeded the treated cells in RPMI 5% FCS and studied their growth for 7 days. Both HT-29 and CACO-2 cells having been treated with 5-FU alone showed poor recovery of cell growth (Fig. 2D and E). HT-29 cells having been treated with M1 or M2 CM showed similar growth recovery as the untreated control while CACO-2 cells treated with M1 CM had a marked reduction in cell growth recovery, with cell count reduced to about 30% of a control after 7 days of recovery (Fig. 2D and E). Interestingly, HT-29 cells having been treated with 5-FU combined with M1 CM (4 h with M1 CM + 24 h combined with 5-FU) recovered their growth to a much greater extent than HT-29 cells treated with 5-FU alone or 5-FU in combination with M2 CM (Fig. 2D). In contrast, CACO-2 cells having been treated with a combination of 5-FU and M1 CM showed poor growth recovery, similar to that of 5-FU treatment alone (Fig. 2E). There was no induction of apoptosis observed in either HT-29 or CACO-2 cells treated with 5-FU (20 μM, 24 h), M1 or M2 CM (28 h) as determined by TUNEL assay (results not shown, n=3–6 independent experiments).

### CM from macrophages of M1 phenotype reduces the 5-FU dependent accumulation of cells in S-phase in HT-29 cells, but not in CACO-2 cells

The cell cycle distribution was analyzed for HT-29 and CACO-2 cells treated with 5-FU, when administered either alone or in combination with either M1 or M2 CM. 5-FU treatment alone, caused accumulation of cells in S-phase and a large reduction of cells in G_2_/M phases in both HT-29 and CACO-2 cells (Table II). In HT-29 cells, treatment with M1 CM caused a substantial decrease of cells in S-phase with an accumulation of cells in G_2_/M and G_0_/G_1_, while treatment with M2 CM had no significant effect on the cell cycle distribution, when compared to control cells (Table II). Combined treatment of HT-29 cells with 5-FU and M1 CM revealed a significantly reduced 5-FU induced accumulation of cells in S-phase and retention of cells in G_2_/M when compared to 5-FU alone, while combined treatment with 5-FU and M2 CM revealed cell cycle distributions similar to that of 5-FU alone (Table II). In contrast, for CACO-2 cells, treatment with M1 or M2 CM did not cause any change in the cell cycle distribution when compared to control cells. Furthermore, the combined treatment of CACO-2 cells with 5-FU and M1 or M2 CM showed a cell cycle distribution similar to that of 5-FU alone, indicating that M1 and M2 CM does not affect the cell cycle to any major extent in CACO-2 cells (Table II).

### CM from macrophages changes the mRNA expression of some 5-FU metabolic genes in HT-29, but is not consistent with reduced cytostatic effect of 5-FU

**Figure 3:**
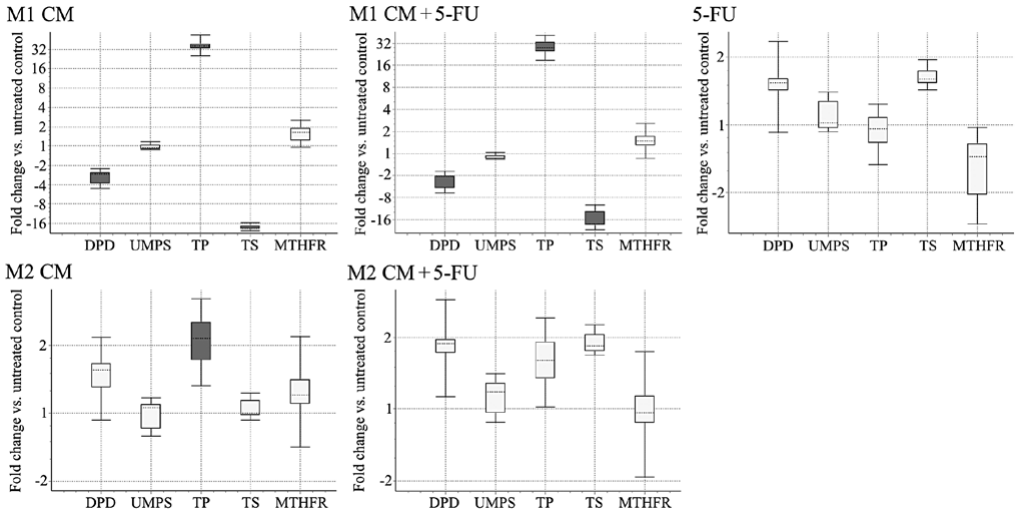
mRNA expression of genes involved in 5-fluorouracil (5-FU) metabolism in HT-29 cells. Cells were treated as indicated with either M1 or M2 conditioned media (CM) for 28 h, 5-FU (20 μM) for 24 h, or combined treatment with M1 or M2 CM and 5-FU (4 h with M1 or M2 CM + 24 h in combination with 5-FU). Results are expressed as fold change of treated cells when compared to control cells and presented in whisker box plots. For each gene analyzed, three independent experiments were performed with three different macrophage batches from different donors. Observe different range of the y-axis between treatments. Gray boxes indicate an up- or downregulation of the mean value, 2-fold or more and white boxes indicate an up- or downregulation of the mean value of <2-fold.

The finding of a reduction in efficacy of 5-FU in HT-29 cells in response to a combined treatment with M1 CM could depend on changes in the cell cycle regulation and/or regulation of the metabolism of 5-FU in these cells. We analyzed the mRNA expression of some key enzymes involved in 5-FU metabolism. In HT-29 cells treated with M1 CM, thymidine phosphorylase (TP) was highly upregulated (32-fold increase), TS was substantially downregulated (16-fold decrease) and dihydropyrimidine dehydrogenase (DPD) was slightly downregulated while uridine monophosphate synthetase (UMPS) and methylenetetrahydrofolate reductase (MTHFR) were unaffected as noted in three independent experiments (Fig. 3). The same expression pattern was seen in HT-29 cells treated with M1 CM in combination with 5-FU. Treatment of HT-29 cells with M2 CM, 5-FU or 5-FU in combination with M2 CM did not change the mRNA expression to any major extent when compared to control. Since the mRNA expression of TS and TP were strongly affected in M1 CM treated HT-29 cells we also examined the mRNA expression of TS and TP in M1 CM treated CACO-2 cells. The same expression pattern as seen in HT-29 cells, although to a lower extent, was also seen in M1 CM treated CACO-2 cells; upregulation of TP (4-fold increase) and downregulation of TS (2-fold decrease) (results not shown).

### CM from macrophages of M1 phenotype affects the mRNA expression of cell cycle regulatory genes in HT-29 and CACO-2 cells

**Figure 4:**
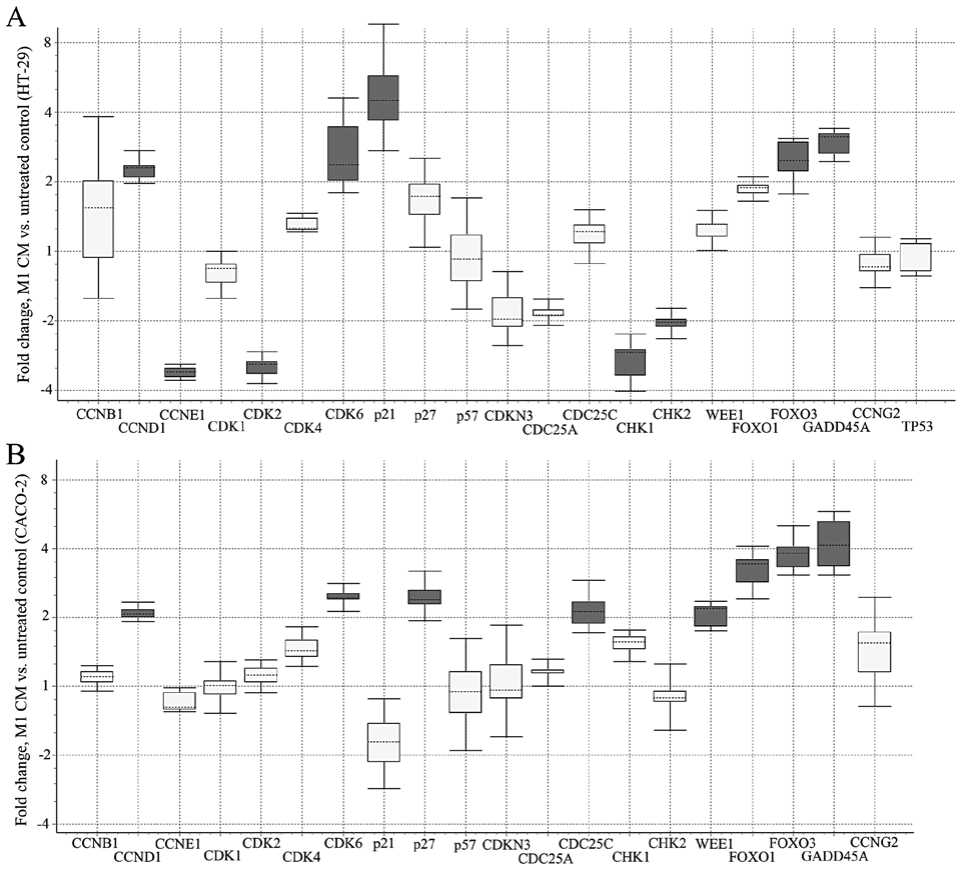
mRNA expression of genes involved in cell cycle regulation in (A) HT-29 cells and (B) CACO-2 cells treated with M1 conditioned media (CM) for 28 h. Results are expressed as fold change in response to M1 CM treatment compared to control and presented in whisker box plots. For each gene analyzed, three independent experiments were performed using three different macrophage batches from different donors. Gray boxes indicate a mean up- or downregulation 2-fold or more and white boxes indicate a mean up- or downregulation <2-fold. CCN, cyclin; CDC, cell division cycle; CDK, cyclin-dependent kinase; CDKN: cyclin-dependent kinase inhibitor, CHK, checkpoint division cycle; FOXO: forkhead box O, GADD: growth arrest and DNA-damage-inducible.

The mRNA expression of cell cycle regulatory genes were analyzed in both HT-29 cells and CACO-2 cells after treatment with M1 CM and the results were compared to an untreated control from either cell line. In HT-29 cells, genes that were upregulated >2-fold when treated with M1 CM were cyclin (CCN)-D1, cyclin-dependent kinase (CDK)-6, forkhead box O (FOXO)-3, growth arrest and DNA-damage-inducible (GADD)-45A and p21. In contrast, CCNE1, CDK2, checkpoint division cycle (CHK)-1 and CHK2 were downregulated 2-fold or more (Fig. 4A). mRNA expression of some of the genes affected by M1 CM in HT-29 cells (CCNE1, CDK2, p21, FOXO3 and GADD45A) were also analyzed in HT-29 cells after treatment with M2 CM, but none of these showed changes in expression when compared to control cells (results not shown). In CACO-2 cells, we observed some differences in mRNA expression of some cell cycle regulatory proteins in response to M1 CM treatment when compared to that seen in HT-29 cells. p21 was not upregulated in CACO-2 cells treated with M1 CM, and there were no downregulation of CCNE1 and CDK2 (Fig. 4B). There was a slight upregulation of cell division cycle (CDC)-25C and WEE1 in CACO-2 cells which was not seen in HT-29 cells, and also a 2-fold or more upregulation in CCND1, CDK6, p27, FOXO1, FOXO3 and GADD45A, being similar to that seen in HT-29 cells. p16 was undetected in both HT-29 and CACO-2 cells, for both untreated and M1 CM treated cells (results not shown). We also examined the mRNA expression of mutated p53 in HT-29 since it has been suggested to mediate resistance to anticancer drugs (28), however, the p53 expression was not affected by M1 CM treatment (Fig. 4A).

### Induced p21 protein expression in HT-29 cells in response to treatment with CM from macrophages of M1 phenotype does not affect the cell cycle or 5-FU cytotoxicity in HT-29 cells

**Figure 5:**
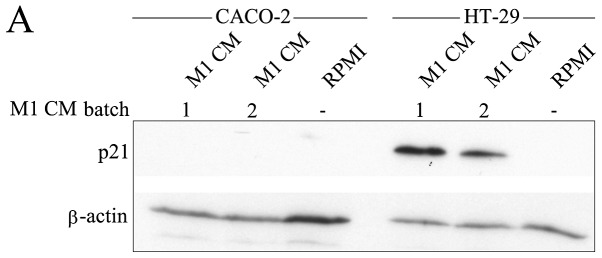

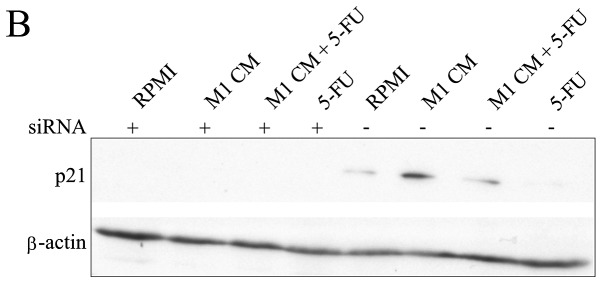
Western blotting of p21. (A) CACO-2 and HT-29 cells treated with M1 conditioned media (CM) for 28 h or untreated controls (RPMI) and subjected to western blotting. Treatment with two different batches of M1 CM are shown in (A) and the results are representative of four different batches of M1 CM. (B) HT-29 cells treated with 30 nM small interfering RNA (siRNA) against p21 (siRNA+) or negative scramble sequence (siRNA-) and further treated with M1 CM for 28 h, 5-fluorouracil (5-FU) (20 μM) for 24 h or M1 CM + 5-FU (4 h with M1 CM + 24 h in combination with 5-FU) and subjected to western blotting. The results in (B) are from one representative experiment out of two. β-actin was used as loading control.

In agreement with the observed mRNA data for p21 (Fig. 4), the expression of p21 protein was induced by M1 CM treatment of HT-29 cells, but not of CACO-2 cells (Fig. 5). To investigate whether the induction of p21 in HT-29 cells was important for cell cycle arrest and 5-FU resistance, we knocked down the p21 expression with siRNA (Fig. 5). Knockdown of p21 did not reveal any changes in cell cycle distribution, cell proliferation or cell growth recovery in response to treatment with M1 CM and 5-FU, in combination or alone, when compared to negative siRNA scramble sequence control (results not shown, n=2 independent experiments).

## Discussion

In this study we have shown that CM from human macrophages of M1 phenotype attenuated the cytotoxic effect of 5-FU in the colon cancer cell line HT-29, but not in the colon cancer cell line CACO-2, and not by CM from the M2 phenotype of macrophages. Treatment of HT-29 cells with 5-FU in combination with M1 CM resulted in growth arrest of either cell line, as expected, because both M1 CM and 5-FU induced growth arrest in HT-29 cells on their own. Interestingly, after removal of M1 CM and 5-FU and continued culture, the HT-29 cells recovered their proliferation to a much larger extent than HT-29 cells having been treated with 5-FU alone or the combined treatment with M2 CM and 5-FU. Although CACO-2 cells exhibited similar growth arrest in response to M1 CM or 5-FU treatment, they did not recover their cell growth after the combined treatment with M1 CM and 5-FU (compare Fig. 2D and E).

5-FU primarily targets proliferating cells, and more specifically cells going through S-phase by depleting dTMP nucleotides thereby leading to failed DNA synthesis (29). Studies have shown that some colon cancer cells can be protected from cytotoxic effects of 5-FU through induction of cell cycle arrest in G_0_/G_1_ and/or G_2_/M (22,30). This seems to be the case for the HT-29 cell line. Thus, HT-29 cells treated with M1 CM were arrested in both G_0_/G_1_ and G_2_/M. Treatment of HT-29 cells with 5-FU induced accumulation of cells in S-phase and this accumulation was significantly reduced in HT-29 cells in response to combined treatment with M1 CM and 5-FU. The cell cycle arrest induced by M1 CM treatment is most likely a strong contributing factor for the attenuated cytotoxic effect of 5-FU seen on HT-29 cells. In support of this conclusion, M1 CM treatment of CACO-2 cells did not induce cell cycle arrest, neither did M1 CM treatment protect CACO-2 cells from the cytotoxic effect of 5-FU.

In an attempt to understand the mechanisms behind the cell cycle regulation, we examined how M1 CM treatment of HT-29 and CACO-2 cells affected the mRNA expression of some genes known to participate in cell cycle regulation. Of the genes analyzed, the mRNA expression of p21 was found to be substantially upregulated in M1 CM treated HT-29 cells while seemingly unchanged in CACO-2 cells. Moreover, the increase in p21 was seen also at the protein level in HT-29 cells, but not in CACO-2 cells. p21 function as an inhibitor of multiple CDKs with the ability to induce cell cycle arrest in both G_0_/G_1_ and G_2_/M (31,32) and an increase in p21 expression has been reported to contribute to 5-FU resistance in cancer cell lines of colon and other origins (33,34). Besides p21 upregulation, M1 CM treatment also induced downregulation of CCNE1 and CDK2 in HT-29, but not in CACO-2. The CCNE1/CDK2 complex is required for G1-S transition, and the complex is blocked by p21 (35).

The upregulation of p21 is presumably mediated via a p53-independent pathway since HT-29 cells has a mutated TP53 gene (R273H mutation) (36). Although the mutated p53 has lost its ability to induce p21 expression, it has been shown to gain functions affecting cell proliferation and resistance to anticancer drugs (28). We therefore examined the mutated p53 mRNA expression in HT-29 cells treated with M1 CM, but no change in expression was observed when compared to control. The FOXO transcription factors can regulate p21 gene expression independently of p53 (37). Both FOXO1 and FOXO3 were upregulated in HT-29 cells when treated with M1 CM and could be factors that induce p21 expression in HT-29 cells. However, FOXO1 and FOXO3 were upregulated also in CACO-2 cells, but without any increase in p21 expression. FOXO transcription factors have also been reported to induce transcription of the cell cycle inhibitors, p27, GADD45A and CCNG2 (38–40). GADD45A and p27 were upregulated in both HT-29 and CACO-2 cells but since CACO-2 cells did not show cell cycle arrest, GADD45A and p27 appear to be less likely mediators of cell cycle arrest in HT-29 cells.

Using p21 knockdown experiments, we investigated whether p21 could be a major contributor to the cell cycle arrest seen in HT-29 cells treated with M1 CM. The M1 CM induced p21 protein expression in HT-29 cells was inhibited by p21 siRNA treatment (Fig. 5). However, the knockdown of p21 did not affect the cell cycle arrest induced by M1 CM, nor did it affect the proliferation of HT-29 cells or the growth recovery after M1 CM, 5-FU or combined M1 CM and 5-FU treatment. These results, therefore, strongly suggest that p21 is not a major contributor to the cell cycle arrest induced by M1 CM.

Since 5-FU needs to be metabolized inside the cell in order to become cytotoxic, we reasoned that M1 CM might also affect the expression of enzymes involved in 5-FU metabolism in a manner that could contribute to an attenuated effect of 5-FU on HT-29 cells.

A series of enzymes are involved in the conversion of 5-FU into its primary active component FdUMP which binds to the nucleotide-binding site of TS and thereby causes the inactivation of this enzyme (24). TS and FdUMP form a complex together with 5,10-methylenetetrahydrofolate (MTHF) and a high concentration of MTHF improves the response of 5-FU, which is the reason why the MTHF precursor, leucovorin, often is used in combination with 5-FU (41). MTHFR is a key enzyme in the folate metabolism that converts MTHF into 5-methyltetrahydrofolate, therefore, high levels of MTHFR could reduce the effect of 5-FU. TP converts 5-FU into 5-fluorodeoxyuridine which is further converted into FdUMP (42). 5-FU can also be converted into fluorouridine triphosphate (FUTP) which contributes to 5-FU toxicity through its incorporation into RNA (43). FUTP is created through phosphorylation of 5-FU to fluorouracil monophosphate, primarily via UMPS which is further phosphorylated to FUTP (44). The rate-limiting catabolic enzyme for 5-FU is DPD that converts 5-FU into dihydrofluorouracil, and high DPD activity can increase resistance to 5-FU (45).

Of the key enzymes in 5-FU metabolism analyzed in HT-29 cells, several were regulated at the mRNA level by M1 CM, but not in a manner that would imply an increase in resistance to 5-FU. Thus, e.g., DPD was downregulated by M1 CM. Furthermore, TP was highly upregulated (32-fold increase) by M1 CM and TS was highly downregulated (16-fold decrease). Both upregulation of TP and downregulation of TS has been reported to increase the cytotoxic effect of 5-FU (46,47), which is the opposite of what we see in M1 CM treated HT-29 cells. In addition, CACO-2 cells, which did not change their responsiveness to 5-FU following M1 CM treatment also showed downregulation of TS and upregulation of TP mRNA. When taken together, the upregulation of TP and the downregulations of TS and DPD all seem unlikely to be responsible for the reduced responsiveness to 5-FU seen in HT-29 cells treated with M1 CM.

In conclusion, our results show that treatment of HT-29 cells with M1 CM attenuates the cytotoxic action of 5-FU and that this effect is mediated by M1 CM-induced cell cycle arrest in G_0_/G_1_ and G_2_/M. We cannot, so far, offer a molecular mechanism of action of this attenuating effect of M1 CM on 5-FU cytotoxicity. Interestingly, this effect could not be mimicked in another colon cancer cell line, CACO-2, albeit the fact that this cell line responded similarly to HT-29 on a single treatment with M1 CM or 5-FU. This lack of mimicry remains to be elucidated. The current findings may imply that the efficacy of 5-FU based chemotherapy for some colon cancer patients could be, in part, dependent on the phenotype of the macrophages residing in the tumor stroma. Further studies along the current line of investigations are warranted in order to optimize chemotherapy in e.g., colon cancer.

## Figures and Tables

**Table I:** Primer sequences used for qPCR.

Gene name	Forward primer sequence 5′→3′	Reverse primer sequence 5′→3′	Genebank accession no.
β-actin	ATTGCCGACAGGATGCAGAA	GCTGATCCACATCTGCTGGAA	NM_001101.3
CCNB1	AACTTTCGCCTGAGCCTATTTT	TTGGTCTGACTGCTTGCTCTT	NM_031966.3
CCND1	CAATGACCCCGCACGATTTC	CATGGAGGGCGGATTGGAA	NM_053056.2
CCNE1	GCCAGCCTTGGGACAATAATG	CTTGCACGTTGAGTTTGGGT	NM_001238.2
CCNG2	TCTGTATTAGCCTTGTGCCTTCT	CCTTGAAACGATCCAAACCAAC	NM_004354.2
CDC25A	GTGAAGGCGCTATTTGGCG	TGGTTGCTCATAATCACTGCC	NM_001789.2
CDC25C	ATGACAATGGAAACTTGGTGGAC	GGAGCGATATAGGCCACTTCTG	NM_001790.3
CDK1	AAACTACAGGTCAAGTGGTAGCC	TCCTGCATAAGCACATCCTGA	NM_001786.4
CDK2	GTACCTCCCCTGGATGAAGAT	CGAAATCCGCTTGTTAGGGTC	NM_001798.3
CDK4	CTGGTGTTTGAGCATGTAGACC	GATCCTTGATCGTTTCGGCTG	NM_000075.3
CDK6	CCAGATGGCTCTAACCTCAGT	AACTTCCACGAAAAAGAGGCTT	NM_001145306.1
CDKN3	TCCGGGGCAATACAGACCAT	GCAGCTAATTTGTCCCGAAACTC	NM_005192.3
CHK1	ATATGAAGCGTGCCGTAGACT	TGCCTATGTCTGGCTCTATTCTG	AF016582.1
CHK2	TTATCTGCCTTAGTGGGTATCCA	CTGTCGTAAAACGTGCCTTTG	NM_001005735.1
DPD	GGCGGACATCGAGAGTATCCT	TTCTTGGCCGAAGTGGAACAC	NM_000110.3
FOXO1	GGATGTGCATTCTATGGTGTACC	TTTCGGGATTGCTTATCTCAGAC	NM_002015.3
FOXO3	CGGACAAACGGCTCACTCT	GGACCCGCATGAATCGACTAT	NM_001455.3
GADD45A	GAGAGCAGAAGACCGAAAGGA	CAGTGATCGTGCGCTGACT	NM_001924.3
GAPDH	CAACAGCGACACCCACTCCT	CACCCTGTTGCTGTAGCCAAA	NM_002046.4
MTHFR	GAGCGGCATGAGAGACTCC	CCGGTCAAACCTTGAGATGAG	NM_005957.4
p21	TTAGCAGCGGAACAAGGAGT	AGCCGAGAGAAAACAGTCCA	NM_000389.4
p27	TAATTGGGGCTCCGGCTAACT	TGCAGGTCGCTTCCTTATTCC	NM_004064.3
p57	GCGGCGATCAAGAAGCTGT	GCTTGGCGAAGAAATCGGAGA	NM_001122631.1
p16	ATGGAGCCTTCGGCTGACT	GTAACTATTCGGTGCGTTGGG	NM_000077.4
POLR2F	ATGTCAGACAACGAGGACAATTT	TTCGGCATTCTCCAAGTCATC	NM_021974.3
RPL37A	ATTGAAATCAGCCAGCACGC	AGGAACCACAGTGCCAGATCC	NM_000998.4
TP	GGTGTGGGTGACAAGGTCAG	GCAGCACTTGCATCTGCTC	NM_001953.4
TP53	CAGCACATGACGGAGGTTGT	TCATCCAAATACTCCACACGC	NM_000546.5
TS	CTGCTGACAACCAAACGTGTG	GCATCCCAGATTTTCACTCCCTT	NM_001071.2
UMPS	GTGTGTGGAGTGCCTTATACAG	CCTTCTACAAGACGCTTAGTTCC	NM_000373.3
WEE1	GACGAAGATGATTGGGCATCC	TGGACTGGAGATCCTTGTTACA	NM_001143976.1

qPCR, quantitative PCR; CCN, cyclin; CDC, cell division cycle; CDK, cyclin-dependent kinase; CDKN, cyclin-dependent kinase inhibitor; CHK, checkpoint division cycle; DPD, dihydropyrimidine dehydrogenase; FOXO, forkhead box O; GADD, growth arrest and DNA-damage-inducible; GAPDH, glyceraldehyde-3-phosphate dehydrogenase; MTHFR, methylenetetrahydrofolate reductase; POLR2F, polymerase (RNA) II (DNA directed) polypeptide F; RPL37A, ribosomal protein L37a; TP, thymidine phosphorylase; TP53, tumor protein p53; TS, thymidylate synthetase; UMPS, uridine monophosphate synthetase.

**Table II:** Cell cycle analysis of HT-29 cells and CACO-2 cells.

Treatment	Cell line	G_0_/G_1_ (%)	S-phase (%)	G_2_/M (%)
RPMI 5% FCS	HT-29	74.1±5.6	17.7±2.9	8.2±3.2
M1 CM	HT-29	82.1±2.2	2.7±0.6^c^	15.2±1.8^c^
M1 CM + 5-FU	HT-29	64.9±7.8	26.5±7.8^d^	8.5±1.8^d^
M2 CM	HT-29	80.2±2.8	13.7±2.0	6.1±1.8
M2 CM + 5-FU	HT-29	64.8±7.8	33.3±5.5^b^	1.9±2.5^b^
5-FU	HT-29	60.5±5.9^a^	38.8±6.5^c^	0.7±0.9^b^
RPMI 5% FCS	CACO-2	44.8±2.4	40.4±0.7	14.8±2.4
M1 CM	CACO-2	49.8±4.3	36.0±4.4	14.2±0.6
M1 CM + 5-FU	CACO-2	45.3±0.8^d^	53.2±3.3^b^	1.5±2.5^c^
M2 CM	CACO-2	43.1±1.7	43.2±2.6	13.6±2.4
M2 CM + 5-FU	CACO-2	49.1±1.4^a^	50.9±1.4^c^	0.0±0.0^c^
5-FU	CACO-2	49.6±0.3^b^	50.4±0.3^c^	0.0±0.0^c^

Cells were treated with either M1 or M2 CM for 28 h, 5-FU (20 μM) for 24 h, or combined treatment with M1 or M2 CM and 5-FU (4 h with M1 or M2 CM + 24 h in combination with 5-FU). Results are shown as mean value ± standard deviation (SD) (HT-29: n≥4 independent experiments, CACO-2: n≥3 independent experiments). At least four (three for CACO-2) different macrophage batches from different donors were used. Unpaired two-sided t-tests

a P<0.05,

b p<0.01,

c p<0.001 compare treatments vs. untreated control (RPMI 5% FCS).

d Indicate statistical significant difference for M1 CM + 5-FU treatment vs. 5-FU treatment using the unpaired two-sided t-test (p<0.05).

FCS, fetal calf serum; CM, conditioned media; 5-FU, 5-fluorouracil.

## Abbreviations

5-FU: 5-fluorouracil
CCN: cyclin
CDC: cell division cycle
CDK: cyclin-dependent kinase
CHK: checkpoint division cycle
CM: conditioned media
CRC: colorectal cancer
DPD: dihydropyrimidine dehydrogenase
FdUMP: fluorodeoxyuridine monophosphate
FOXO: forkhead box O
FUTP: fluorouridine triphosphate
GADD: growth arrest and DNA-damage-inducible
MTHF: methylenetetrahydrofolate
MTHFR: methylenetetrahydrofolate reductase
TAM: tumor-associated macrophages
TP: thymidine phosphorylase
TS: thymidylate synthetase
UMPS: uridine monophosphate synthetase
